# Secondary Amyloidosis Presenting as Ischemic Proctitis

**DOI:** 10.1155/2021/6663391

**Published:** 2021-04-08

**Authors:** Saad Hashmi, Assad Munis, Ryan T. Hoff, Hymie Kavin, Eli D. Ehrenpreis

**Affiliations:** ^1^Advocate Lutheran General Hospital, Department of Internal Medicine, Park Ridge, IL, USA; ^2^Advocate Lutheran General Hospital, Department of Internal Medicine, Division of Gastroenterology, Park Ridge, IL, USA

## Abstract

A 49-year-old man presented with abdominal pain and rectal bleeding for two days associated with a 50-pound unintentional weight loss. History was notable for hypertension, chronic kidney disease, obesity, gout, and acute cholecystitis status post cholecystectomy. Computed tomography (CT) of the abdomen and pelvis showed rectal wall thickening. Colonoscopy showed proctitis with superficial ulcerations. In the setting of renal insufficiency, malabsorption, and low-voltage QRS complexes on electrocardiogram (ECG), amyloidosis was considered in the differential diagnosis. Rectal and renal biopsies with subsequent retrospective staining of gallbladder tissue confirmed amyloid deposition. Gastrointestinal involvement of amyloidosis is relatively uncommon. Particularly, amyloid deposition in the gallbladder and rectum is very rare. The development of AA amyloidosis in our patient may have been related to gout, obesity, and the presence of a heterozygous complex variant for the MEFV (familial Mediterranean fever) gene. Awareness of this atypical presentation of amyloidosis is important, as additional staining of biopsy samples is necessary, and diagnosis allows for directed treatment.

## 1. Introduction

Amyloidosis is an infiltrative disorder characterized by extracellular deposition of insoluble proteins in various tissues. These insoluble proteins are folded in such a manner that they cannot be degraded by cellular proteases, resulting in accumulation of amyloid fibrils. Subsequently, the structure and function of organs that contain these deposited proteins are impaired. The most common form of systemic amyloidosis is primary amyloidosis (AL amyloidosis), which is caused by immunoglobulin light chain deposition. Secondary amyloidosis (AA amyloidosis) is caused by deposition of serum amyloid A protein (SAA), an acute-phase reactant produced in response to inflammation. Thus, AA amyloidosis is most often seen in chronic states of inflammation. Secondary amyloidosis commonly affects the kidneys, skin, liver, and spleen. Involvement of the gastrointestinal (GI) tract occurs in only about 3–8% of all cases of AA amyloidosis. Within the GI tract, amyloid deposits confirmed by histology are most often detected in the small intestine (50%), stomach (44%), colon (32%), esophagus (12%), and rectum (8%) [1].

Gastrointestinal manifestations of amyloidosis include abdominal pain, weight loss, malabsorption, dysmotility, hepatomegaly, splenomegaly, jaundice, and GI bleeding. Amyloid deposition in the GI tract often occurs in the muscularis mucosa in close proximity to blood vessels and nerves. Gastrointestinal bleeding occurs secondary to vascular fragility and mucosal ulcerations. Compared with GI amyloidosis, ischemic colitis more often presents with abdominal pain associated with GI bleeding and commonly affects areas of the colon that have limited collateralization, rarely affecting regions with dual blood supply, such as the rectum [2]. We report a case of ischemic proctitis as the presentation of secondary amyloidosis.

## 2. Case Presentation

**Figure 1:**
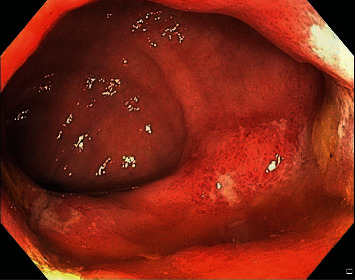
Colonoscopy showing rectum with proctitis, characterized by erythema and scattered superficial ulcerations.

**Figure 2:**
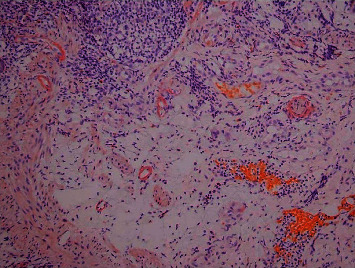
Rectal biopsy with Congo Red stain. Amyloid deposition is seen in the rectal mucosal vessel walls and lamina propria.

**Figure 3:**
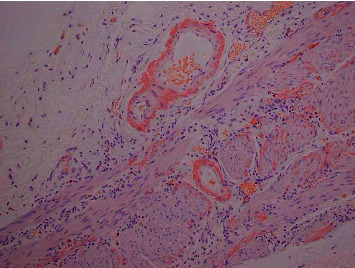
Postoperative histology of gallbladder with Congo Red stain. Amyloid deposition is seen in the gallbladder vessel.

A 49-year-old male presented with two days of rectal bleeding in the setting of two months of acute on chronic diffuse abdominal pain and anorexia. Comorbid conditions included hypertension, obesity, gout, and chronic kidney disease. He admitted to malaise, nausea, and vomiting. The patient's baseline weight was around 271 lbs with a BMI of 42. However, upon presentation, his weight was down to 216 lbs accounting for a 55 lbs unintentional weight loss over 6 months. Seven months prior, he developed acute cholecystitis associated with a gallstone and underwent cholecystectomy. He was afebrile and normotensive. His abdominal exam revealed diffuse tenderness to palpation. Laboratory studies showed a microcytic anemia with hemoglobin of 10.8 g/dL, creatinine 2.69 mg/dL, urine protein 65 mg/dL, albumin 2.6 g/dL, an isolated elevation of serum alkaline phosphatase (234 IU/L), hyperglobulinemia, and an elevated gamma-glutamyltransferase. An electrocardiogram showed normal sinus rhythm with low-voltage QRS complexes. A CT scan of the abdomen and pelvis revealed rectal wall thickening and perirectal fat infiltration. An endoscopy to evaluate upper abdominal pain revealed superficial antral erosions and dilated duodenal lacteals, with biopsies showing normal-appearing gastric and duodenal mucosa. Colonoscopy showed distal proctitis with superficial ulcerations (Figure 1). Hematoxylin and eosin staining showed acute inflammation, mucosal erosion, hyalinizing fibrosis of the lamina propria, and atrophic colonic crypts, compatible with ischemic colitis. In the setting of renal insufficiency, malabsorption, and low-voltage QRS complexes on electrocardiogram (ECG), amyloidosis was considered in the differential diagnosis. Thus, Congo Red staining of rectal biopsies was performed, which revealed amyloid deposition in the rectal mucosal vessel walls and lamina propria (Figure 2). Retrospective staining of the gallbladder vessels also demonstrated amyloid deposition (Figure 3). Biopsies of the gastric body and antrum and duodenum were positive for amyloid in walls of small blood vessels and in lamina propria. Abdominal fat pad biopsy was inconclusive for definitive amyloidosis. A bone marrow biopsy revealed a small amount of amyloid in the blood vessels. A renal biopsy then revealed glomerular, vascular, and interstitial amyloid A protein deposition. Mass spectrometry analysis showed amyloid A material consistent with secondary amyloidosis. Serum protein electrophoresis (SPEP) showed normal free light chain ratio, which ruled out primary amyloidosis.

To evaluate the cause for the patient's secondary amyloidosis, the patient was assessed for MEFV gene mutations, which can cause familial Mediterranean fever. The patient was found to be heterozygous for a complex variant of the MEFV gene consisting of two nucleotide changes, c.1105 C > T and c.1223 G > A, on the same chromosome. This variant has been historically associated with familial Mediterranean fever (FMF) in the medical literature [3–5]. However, it has also been observed at a higher frequency in healthy carriers than affected individuals [6]. Genotype-phenotype analyses indicate that the majority of patients who have FMF and carry this complex variant have a wide range of clinical symptoms associated with atypical FMF [7].

Further testing was negative for rheumatoid factor and ANA. The patient's presentation and laboratory findings did not suggest any autoimmune arthritides, vasculitides, inflammatory bowel disease, neoplasms, or chronic infections. Although no definitive cause for secondary amyloidosis was identified, we postulate that the patient's history of gout, obesity, and presence of a heterozygous complex variant for the MEFV gene may have contributed to the development of AA amyloidosis.

Given the patient's poor nutritional status and weight loss, a naso-jejunal tube was placed via enteroscopy which was removed a few weeks later once the patient was able to tolerate oral intake. Therapy was directed towards treating the possible underlying causes for AA amyloidosis. Colchicine was started to treat gout and any inflammation related to the patient's polymorphisms associated with familial Mediterranean fever. Unfortunately, the patient's renal disease progressed, requiring hemodialysis. With worsening orthostatic hypotension leading to recurrent hospitalizations and eventually precluding continued hemodialysis, the patient was transitioned to hospice care.

## 3. Discussion

This unique case emphasizes the clinical importance of secondary amyloidosis as a systemic disease and its potential for unusual presentations. Clinical manifestations of amyloidosis result from the deposition of amyloid protein in the mucosal and submucosal linings of various body organs. Gastrointestinal amyloidosis can result in a variety of seemingly unrelated manifestations, including chronic dysmotility, protein-losing gastroenteropathy, malabsorption, or nonocclusive colonic ischemia [8]. The wide spectrum of clinical manifestations can make amyloidosis a mimic for colorectal cancer, as patients often present with anemia, lower GI bleed, and/or weight loss [9]. Amyloid deposition in the gallbladder resulting in acute cholecystitis is uncommon and has been reported in a small number of cases [10, 11]. This case reinforces the necessity of forming an extensive differential diagnosis to avoid a misdiagnosis or delayed treatment.

Endoscopic manifestations of amyloidosis include erythema, focal erosions or ulcerations, friability, or plaque-like mucosa. Up to 32% may have normal endoscopic appearance [8]. As the endoscopic findings of amyloidosis are nonspecific and often absent altogether, early diagnosis is a challenge and endoscopy alone is insufficient to diagnose AA amyloidosis [12]. In our patient with proctitis in the absence of hemodynamic compromise, acute vascular occlusion, or other cause of rectal ulceration, we requested Congo Red staining of rectal biopsy samples.

Ischemic proctitis is often associated with radiation therapy, aortic surgery, or other vascular interventions. Data are lacking regarding the prevalence of lower gastrointestinal bleeding as a result of amyloidosis, resulting in limited evidence-based guidance regarding optimal evaluation and management. However, amongst patients with GI amyloidosis, between 25 and 45% present with gastrointestinal bleeding [1]. In cases of suspected GI amyloidosis, rectal biopsies should be considered, as the sensitivity is approximately 75 to 85 % compared with other sites [13]. Awareness of this presentation of amyloidosis is important, as clinicians must request additional staining of biopsy samples if there is a high index of suspicion, as it is not performed routinely. Rectal biopsies may confirm the diagnosis of amyloidosis when fat pad biopsies are negative or inconclusive, as in our patient.

In order to treat AA amyloidosis, it is important to first identify and control the underlying inflammatory disease, which results in decreased production of SAA protein. An association of patients with heterozygous MEFV mutations developing familial Mediterranean fever-related AA amyloidosis has been suggested [14, 15]. Additionally, there are a few reported cases of gout-associated AA amyloidosis and many of the published cases included renal involvement [16, 17]. Based on a few case reports, there is a hypothesis that the low-grade chronic inflammation seen in obesity may contribute to the development of AA amyloidosis [18, 19]. As a result, it was suspected obesity, gout, and the presence of a heterozygous MEFV gene mutation contributed to our patient's AA amyloidosis. Several treatment options are available, depending on the underlying disease. Colchicine may be used to treat FMF or gout to help reduce the extent of proteinuria due to renal amyloidosis, and hence colchicine was used for treatment in our patient [20]. Cytotoxic and immunosuppressive agents such as azathioprine, methotrexate, and cyclophosphamide have been shown to be helpful in AA amyloidosis [21]. For rheumatoid arthritis, ankylosing spondylitis, and psoriatic arthritis, biologics with activity against proinflammatory cytokines (TNF-alpha, IL-1 beta, and IL-6) may reduce the risk of developing amyloidosis [22]. Without treatment, individuals with AA amyloidosis have a significant risk of mortality due to end-stage renal disease, infection, GI bleed, heart failure, and/or bowel perforation.

## 4. Conclusion

We describe a patient with rectal bleeding, abdominal pain, and unintentional weight loss, who was found to have ischemic proctitis and malabsorption due to AA amyloidosis. Subsequent evaluation confirmed amyloid deposition in the gallbladder, gastric body, duodenum, and kidneys. Amyloidosis involvement of the gallbladder is very rare; amyloidosis associated with acute cholecystitis is particularly rare. We believe the development of AA amyloidosis in our patient was related to gout, obesity, and genetic factors. Awareness of this atypical presentation of amyloidosis is important, as additional staining of biopsy samples is necessary, and diagnosis allows for directed treatment.
